# Aggression and spatial positioning of kin and non-kin fish in social groups

**DOI:** 10.1093/beheco/arad036

**Published:** 2023-05-12

**Authors:** Aneesh P H Bose, Johanna Dabernig-Heinz, Jan Oberkofler, Lukas Koch, Jacqueline Grimm, Kristina M Sefc, Alex Jordan

**Affiliations:** Department of Wildlife, Fish & Environmental Studies, Swedish University of Agricultural Sciences (SLU), Skogsmarksgränd, 90183, Umeå, Sweden; Department of Collective Behaviour, Max Planck Institute of Animal Behavior, Universitätsstraße 10, 78464, Konstanz, Germany; Centre for the Advanced Study of Collective Behaviour, University of Konstanz, Universitätsstraße 10, 78464, Konstanz, Germany; Department of Biology, University of Konstanz, Universitätsstraße 10, 78464, Konstanz, Germany; Institute of Biology, University of Graz, Universitätsplatz 2, 8010, Graz, Austria; Department of Collective Behaviour, Max Planck Institute of Animal Behavior, Universitätsstraße 10, 78464, Konstanz, Germany; Centre for the Advanced Study of Collective Behaviour, University of Konstanz, Universitätsstraße 10, 78464, Konstanz, Germany; Department of Biology, University of Konstanz, Universitätsstraße 10, 78464, Konstanz, Germany; Department of Collective Behaviour, Max Planck Institute of Animal Behavior, Universitätsstraße 10, 78464, Konstanz, Germany; Centre for the Advanced Study of Collective Behaviour, University of Konstanz, Universitätsstraße 10, 78464, Konstanz, Germany; Department of Biology, University of Konstanz, Universitätsstraße 10, 78464, Konstanz, Germany; Institute of Biology, University of Graz, Universitätsplatz 2, 8010, Graz, Austria; Institute of Biology, University of Graz, Universitätsplatz 2, 8010, Graz, Austria; Department of Collective Behaviour, Max Planck Institute of Animal Behavior, Universitätsstraße 10, 78464, Konstanz, Germany; Centre for the Advanced Study of Collective Behaviour, University of Konstanz, Universitätsstraße 10, 78464, Konstanz, Germany; Department of Biology, University of Konstanz, Universitätsstraße 10, 78464, Konstanz, Germany

**Keywords:** cichlid, contest behavior, genetic relatedness, group living, kin discrimination, kin selection, sex differences, within-group competition

## Abstract

Group-living animals are faced with the challenge of sharing space and local resources amongst group members who may be either relatives or non-relatives. Individuals may reduce the inclusive fitness costs they incur from competing with relatives by either reducing their levels of aggression toward kin, or by maintaining physical separation between kin. In this field study, we used the group-living cichlid *Neolamprologus multifasciatus* to examine whether within-group aggression is reduced among group members that are kin, and whether kin occupy different regions of their group’s territory to reduce kin competition over space and local resources. We determined the kinship relationships among cohabiting adults via microsatellite genotyping and then combined these with spatial and behavioral analyses of groups in the wild. We found that aggressive contests between group members declined in frequency with spatial separation between their shelters. Female kin did not engage in aggressive contests with one another, whereas non-kin females did, despite the fact these females lived at similar distances from one another on their groups’ territories. Contests within male–male and male–female dyads did not clearly correlate with kinship. Non-kin male-male and male–female dyads lived at more variable distances from one another on their territories than their corresponding kin dyads. Together, our study indicates that contests among group members can be mediated by relatedness in a sex-dependent manner. We also suggest that spatial relationships can play an important role in determining the extent to which group members compete with one another.

## Teaser

Group-living animals must share space and resources with group mates, who can be either kin or non-kin, and it is often unclear how competitive or cooperative group members should be. In a group-living cichlid, we show that co-habiting females are less aggressive to their female kin (relative to non-kin) despite living at equivalent distances to one another. This pattern was not detected among co-habiting males, revealing that kin-directed social behavior can differ between the sexes.

## INTRODUCTION

Animals that live in groups often face conflicts when sharing limited resources with their group members (Krause and Ruxton 2002; Hardy and Briffa 2013; Ward and Webster 2016). How these conflicts are resolved can have far-reaching fitness consequences, especially when within-group competition places relatives in opposition to one another. Here, the competitive traits that individuals express, such as aggression, should be influenced by the direct fitness payoffs of winning a contest, but also by the indirect costs associated with reducing the success of their competitors who might be kin (Pizzari et al. 2015). One way for individuals to navigate this problem is by modulating aggression in accordance with their genetic relatedness to competitors, a form of kin discrimination (Giron et al. 2004). For example, in red junglefowl, *Gallus gallus*, male group members competing for mating access to females will exhibit less aggression toward related competitors than unrelated competitors (Tan et al. 2016). Doing so can reduce the indirect fitness costs associated with impairing the success of relatives.

Another way for group-living animals to navigate the challenge of competing with related and unrelated individuals is to find means of lessening competition with kin. For example, individuals may use different resources from their relatives, thereby reducing direct kin competition, or they might maintain physical distance from their relatives to minimize local competition for resources. The idea that individuals can coordinate their spatial arrangements based on relatedness to one another is typically thought of in terms of kin aggregations and kin avoidance (Hatchwell 2010). Kin can form cooperative coalitions (e.g., lions, *Panthera leo*, Packer et al. 1991), lek in close proximity (e.g., black grouse, *Tetrao tetrix*, Höglund et al. 1999), disperse to avoid competition with relatives (Hamilton and May 1977), or disperse to avoid inbreeding (Pusey and Wolf 1996; Szulkin and Sheldon 2008). In the West African cichlid, *Pelvicachromis taeniatus*, subadults avoid their kin when exploring novel environments, and this is interpreted as a means to reduce kin competition over resources or mates (Vitt et al. 2020). Alternatively, in Columbian ground squirrels, *Urocitellus columbianus*, females make dispersal decisions that help them find less competitive environments to settle in, but they maintain close proximity to their kin, which provides them with inclusive fitness benefits (Arnaud et al. 2012; Dobson et al. 2012).

For animals that live in groups, group members can be both competitors and cooperators. The extent to which group members aggressively compete, or refrain from competing, with one another may depend, in part, on the relatedness structure of the local competitive arena. Here, we suggest that both competitive interactions and the spatial positioning of individuals within a social group can be modulated by kinship. To date, numerous studies have examined the costs and benefits of living at different spatial positions within a group, often by comparing edge versus center positions. Edge and center locations are often associated with different feeding opportunities, predation risks, and levels of social competition, which can result in individuals of differing ages, sexes, and ranks occupying different locations (Hirsch 2007; Hirsch 2011; Heesen et al. 2015; Teichroeb et al. 2015). Different positions in a group may also be more or less beneficial depending on one’s relative proximity to kin or non-kin, particularly as kin neighbors are expected to compete less with one another (Humphries et al. 2021). Yet, the role of kinship in determining how individuals position themselves within a group or a territory has received scant attention to date.

The degree to which kinship influences an individual’s social behavior can also be expected to differ between males and females. One way for this to occur is if either males or females are socially dominant such that subordinates avoid aggressive confrontations with them (e.g., if the costs of contest escalation are high, Willisch and Neuhaus 2010). Since subordinates only rarely employ aggression toward dominants, their kinship to dominants would have little scope to influence aggression. Instead, subordinates may form queues within the hierarchy to await a breeding position, in which case subordinates may compete amongst each other for rank, and dominants may express nepotism in favor of kin over non-kin subordinates. Such discrimination occurs in the southern pied babbler, *Turdoides bicolor*, where dominant males preferentially expel unrelated subordinate males from their groups, thereby giving their own male relatives priority ranks within the social queue, but this does not occur among females (Nelson-Flower and Ridley 2016). Another way for sex differences to arise is if each sex experiences a different “scale of competition” (see Frank 1998). Both males and females can engage in intrasexual competition, and if the individuals that one sex competes with are more likely to be kin than the individuals that the other sex competes with, then kin competition could restrict the scope for relatedness to affect social interactions in the former more so than in the latter (West et al. 2001; West et al. 2002). The notion that competition among kin can work against the benefits of assisting kin is often applied during theoretical examinations of the evolution of indiscriminate altruism in viscous populations, but it can also be applicable in cases where kin are recognized and discriminated (Johnstone and Cant 2008). A sex difference in how related individuals are to their competitors may arise for example through sex-biased dispersal, which causes the more philopatric sex to be more related to their nearby same-sex social partners (Johnstone and Cant 2008; Bose, Koch, et al. 2022). Systems where group members vary in relatedness, have stable spatial arrangements, and display conspicuous contest behaviors are therefore ideal for empirically examining how relatedness influences within-group social dynamics.

In this study, we combined behavioral scoring and microsatellite genotyping of wild individuals of a social shell-dwelling cichlid fish, *Neolamprologus multifasciatus*. We examined whether individuals reduce within-group competition with their kin by modulating aggression toward group members according to their relatedness and/or by maintaining physical separation with their kin. *Neolamprologus multifasciatus* is endemic to Lake Tanganyika, East Africa, and lives in groups that can consist of multiple males, females, and juveniles, with the largest male per group being socially dominant (Kohler 1998; Bose, Koch, et al. 2022; Bose, Dabernig-Heinz, et al. 2022). Groups contain a mixture of related and unrelated individuals, though female-biased dispersal in this system means that, on average, males are more related to other males in their groups than females are to each other (Bose, Koch, et al. 2022; Bose, Dabernig-Heinz, et al. 2022). Reproductive skew is high among males, with the dominant male acquiring nearly all the reproduction that occurs in his group (Bose, Dabernig-Heinz, et al. 2022). Thus, a male’s reproductive success is closely linked to his ability to achieve and retain a dominant position. Each group defends a territory on the sandy lake floor that contains crucial resources, namely empty gastropod shells that the fish use as shelters and as brood chambers to raise their offspring (Kohler 1998; Schradin and Lamprecht 2000; Schradin and Lamprecht 2002; Jordan et al. 2016; Bose et al. 2021). Individuals compete over space as well as access to these non-sharable resources, which can result in aggressive interactions among group members (Jordan et al. 2016; Bose et al. 2021). Furthermore, a group’s territory space is partitioned into largely non-overlapping sub-territories; each sub-territory is controlled by a different adult and forms a separate piece of the mosaic that is their group’s territory (Schradin and Lamprecht 2002; Bose et al. 2021). Fish that live close to one another in a group may be more in conflict with one another over local shells than fish that live on opposite sides of the territory. Fish that are close neighbors may also compete for foraging positions in the water column. The spatial structure of *N. multifasciatus* territories therefore also provided us with the opportunity to examine whether kin relationships are reflected in the relative positioning of individuals. We considered the possibility that kin may space themselves further apart across their group’s territory to lessen their competition over space and resources, but we also considered the possibility that kin may cluster more tightly together within their group’s territory if doing so allows them to jointly defend space and resources more successfully than on their own.

## METHODS

### Study system

*Neolamprologus multifasciatus* is a small-bodied, Lamprologine cichlid that lives in social groups on the floor of Lake Tanganyika in regions that are covered in dense accumulations of empty gastropod shells (i.e., so-called “shell beds”). Shell beds are home to a rich diversity of cichlid fishes (Koblmüller et al. 2007), some of which utilize the empty shells as resources, such as *N. multifasciatus*, while others are predatory and use the shell beds as hunting grounds. Each *N. multifasciatus* group jointly guards a territory on the lake floor, which contains a collection of empty gastropod shells that the fish have excavated from the sediment. *Neolamprologus multifasciatus* territories tend to be small, rarely exceeding 30 by 30 cm in the wild (Bose et al. 2021; personal observations, A.B. and A.J.). The average group size in the wild is approximately six fish and can range up to 22 individuals (Bose, Koch, et al. 2022). Each group member possesses one “home shell” in their sub-territory, which they return to regularly and where females will lay eggs and care for offspring (Gübel et al. 2021). These home shells form the center points of each group members’ sub-territory, yet how each group member chooses their home shell and its location within the wider territory is unknown. Aggression among group members is thought to be driven by the supply of shells that each group controls (Kohler 1998; Bose et al. 2021; Gübel et al. 2021). That is, the number of shells is limited, but dominant males and females each benefit individually if they control more shells, either because it allows them to attract more mates or because it provides space for their offspring to reside in respectively. Thus, *N. multifasciatus* live in tight-knit social groups on the lake floor, but they also frequently engage in contests with one another as they vie for space and resources.

*Neolamprologus multifasciatus* display female-biased dispersal, with females being more likely than males to emigrate from a group, and also to travel farther when they move (Bose, Koch, et al. 2022). A previous population genetics study showed that in *N. multifasciatus* groups, a fair percentage (~22%) of co-habiting male–male dyads have relatedness coefficients in the range of first-grade relatives, with the median around the level of first cousins (Bose, Koch, et al. 2022). Relatedness coefficients between females are closer to zero, with fewer closely related dyads (Bose, Koch, et al. 2022). Juveniles that are raised on a territory together are on average related to one another at the level of half-siblings (though they can range from unrelated to full siblings), which is consistent with most juveniles sharing a single father (the dominant male) but having different mothers (Bose, Dabernig-Heinz, et al. 2022). It is currently unknown whether *N. multifasciatus* uses any particular mechanisms of kin recognition (e.g., familiarity-based cues or phenotype matching, see Penn and Frommen 2010), though kin recognition has been demonstrated in other cichlid fishes to date (e.g., phenotype matching in *Neolamprologus pulcher*, Le Vin et al. 2010, and *Pelvicachromis taeniatus*, Thünken et al. 2007).

### Field sample collection

Between September and October 2019, we identified all *N. multifasciatus* territories in a study quadrat on a shell bed in the south of Lake Tanganyika, Zambia (8°42ʹ49.0″ S 31°07ʹ22.9″ E). The quadrat measured approximately 10 × 10 m, was located at a depth of 10–11 m, and contained over 120 *N. multifasciatus* territories (see Bose, Dabernig-Heinz, et al. 2022; Bose, Koch, et al. 2022 for details). While on SCUBA, we positioned downward facing video cameras approximately 50 cm above 22 territories to film the social interactions among their group members (using GoPro Hero 7 cameras, set to 1080p resolution, 30 fps, and a “linear” field of view to reduce distortion along the edges of the camera’s view). These groups each contained one dominant male and at least one adult female, though some groups also contained additional adult fish. The cameras recorded for at least 50 min each. After each recording, a buddy pair of divers systematically sampled all group members from each territory. *N. multifasciatus* hide within their home shells when approached by a diver, which means that the fish can be captured by picking up the shells containing the hiding fish. While underwater, the fish were extracted from their shells using custom-made extraction chambers, which allow the fish to swim out of their shells naturally, but then hinder them from re-entering (see Supplementary Figure S1 for details). The fish were then sedated with clove oil, sexed by inspecting their urogenital papillae, measured for standard length using calipers (to the nearest 0.1 cm, SL), and recorded as either an adult or a juvenile based on the presence of distinct banding patterns on the sides of their bodies, which denote sexual maturity (Kohler 1998). We also fin-clipped fish that were larger than 1.7 cm in SL on their anal fins (taking at most 2 × 2 mm of tissue). Smaller fish were euthanized with an overdose of clove oil and sampled whole because of the relatively large amount of fin tissue that clipping would have removed. All tissue samples were stored in 99% ethanol for microsatellite genotyping. Importantly, during the sampling, we carefully recorded the location on the territory where each fish was captured. By combining this positional information with their body sizes and sex, we were able to match the identities of the fish in the videos with their tissue samples. This work was carried out with permission from the Fisheries Department of Zambia under study permits issued by the government of Zambia (No. G7067690 and C3195368).

### Behavioral scoring

A single observer who was blind to the kinship among the group members (see below) used the software BORIS (Friard and Gamba 2016) to score contest behaviors that the adult *N. multifasciatus* individuals visible in each video performed toward one another (see Table 1 for behavioral ethogram). We omitted the first 10 min of footage after camera setup, and then scored behavior from the following 40-min time window. Similar time durations have been used by previous studies to capture patterns of social interactions in *N. multifasciatus* groups (e.g., Bose et al. 2021). In total, we scored contest behavior for 22 dominant males, 9 subordinate males, and 38 females. Only within-group aggression was scored. Both aggression and submission are used to settle within-group contests, and so both kinds of behavior were scored (following Jordan et al. 2016; Bose et al. 2021). For each interaction, we specifically recorded which fish was the actor and which was the recipient. This way all the contest behaviors exhibited by particular dyads of fish could be tallied. Periodically, predatory cichlids will swim through the camera’s field of view, close to the territory, causing all group members to temporarily hide in their shells. We used BORIS to record the cumulative duration of these interruptions for each group separately to better account for the time windows during which group members had the opportunity to interact with one another. Lastly, the scorer also took the pixel coordinates of each fish’s home shell and calculated the pairwise distances between all the home shells in each group, with the aid of a ruler placed within the camera’s field of view for scale.

**Table 1:** Ethogram used to score contest behaviors of *Neolamprologus multifasciatus* individuals

Behavior	Description
*Aggression*	
Frontal display	The focal fish faces another fish and spreads its opercula and pectoral fins. Often associated with forward and backwards movements of the body, and/or a rigid body position.
Lateral display	The focal fish positions its body laterally with another fish and adopts a rigid body. Often accompanied by the focal fish thrashing its caudal fin toward the opponent.
Bite/chase/ram	The focal fish accelerates toward another fish and typically makes contact.
Mouth wrestle	The focal fish locks jaws with another fish and they push against each other.
*Submission*	
Submissive display	The focal fish positions its body laterally to another fish and shows its belly. Often accompanied by body quivers.
Flee	The focal fish accelerates away from another fish, often entering into an empty gastropod shell for shelter.
*Miscellaneous*	
Shell hiding	The focal fish hides in an empty gastropod shell for a duration of time.

This ethogram was derived from one in Sopinka et al. (2009), which was originally developed for a related cichlid, *Neolamprologus pulcher*.

### Microsatellite genotyping and relatedness estimation

The current study used microsatellite data that were obtained as part of several other studies (Bose, Koch, et al. 2022; Bose, Dabernig-Heinz, et al. 2022). In the lab, DNA was extracted from fin clips using a standard Chelex protocol (Walsh et al. 1991). All individuals were genotyped at 20 microsatellite loci divided into three multiplexes (see Supplementary Table S1 for details on marker polymorphism). We used 3 µL of Qiagen Type-it Multiplex PCR Master Mix for the multiplex PCRs, along with 1 µL of template DNA, and 0.5 µL of primer mix (see Supplementary Table S1 for concentrations). Total PCR volume was 5.5 µL, and each forward primer was labeled with one of the fluorescent dyes HEX, FAM, NED, ATTO550 and ATTO565. We used the following PCR program settings: denaturation at 95 °C for 5 min, followed by 30 cycles at 95 °C for 30 s, annealing at 55 °C (for multiplex1), 54 °C (for multiplex 2), or 53 °C (for multiplex 3) for 90 s, extension at 72 °C for 30 s, and a final extension at 60 °C for 30 min. We scored allele sizes against an internal standard (GeneScan 500 LIZ, Applied Biosystems) in an automatic sequencer (3130xL Genetic Analyzer, Applied Biosystems) and GeneMapper software (v 3.7, Applied Biosystems).

We estimated population allele frequencies in CERVUS (v 3.0.7; Kalinowski et al. 2007). We used a set of adult fish (*N* = 233) sampled from the same study quadrat that we also used to estimate background allele frequencies in Bose, Dabernig-Heinz, et al. (2022) and Bose, Koch, et al. (2022). The markers were highly polymorphic with an average of 16.4 alleles per locus, a mean observed heterozygosity of 0.74, and all markers adhered to Hardy–Weinberg equilibrium (Supplementary Table S1).

We used the program ML-Relate (Kalinowski et al. 2006) to estimate the level of kinship for each dyad of cohabiting adults. ML-Relate uses individual multi-locus genotypes along with population allele frequencies to calculate maximum likelihood estimates for different relationships: “unrelated,” “half-sibling,” “full-sibling,” and “parent-offspring.” It also uses simulations to test which of these genealogical relationships are more likely than others for each dyad. For our purposes, we used ML-Relate to categorize the genetic relationship between each dyad of fish as either “unrelated” or “related,” which was done based on whether the “unrelated” category could be rejected with 95% confidence. This allowed us to identify dyads of fish that were likely to be related to one another *at least* at the level of half-siblings.

We chose a categorization threshold of at least half siblings based on the harem polygyny mating system of *N. multifasciatus* (Bose, Dabernig-Heinz, et al. 2022). The offspring produced in each *N. multifasciatus* group are typically either siblings or half-siblings, that is, they share the same father but may have different mothers (Bose, Koch, et al. 2022). Thus, fish dyads in our study classified at least as being half-siblings were also likely to have grown up in the same group or in close proximity. The use of half-siblings as a categorization threshold, or rather a relatedness threshold of *r* = 0.25, was additionally supported by the observation of bimodality in the distributions of relatedness estimates between group members with the two peaks being separated by *r* = 0.25 (see Supplementary Figures S2–S4).

### Statistical analyses

We divided the fish that were living in the same groups together into dyads. These were categorized according to sex pairing, that is, female–female, female–male, and male–male, and also according to kinship assignment, that is, related and unrelated (creating six combinations in total). We first fit a generalized linear mixed effects model (GLMM, using the R package “glmmTMB,” Brooks et al. 2017) assuming a negative binomial error distribution to test whether the frequency of contest behaviors expressed in each dyad was correlated with its sex pairing (three-level categorical: female–female, female–male, male–male), its kinship assignment (two-level categorical: related, unrelated), and the distance separating the home shells of the fish in the dyad (cm, log-transformed). Distance was included in the model because the physical separation between territorial animals can have a strong influence on their rates of interaction (e.g., Viblanc et al. 2016). We also included the time window (measured in BORIS at a resolution of 0.1 s, but then converted into minutes) when the fish were not jointly hiding in their shells from predators as a model offset (log-transformed) to account for variation across groups in the time available for contest interactions to take place between group members. The interaction terms between distance and the other two predictor variables were tested with a likelihood ratio test, but did not significantly improve model fit and were therefore not included in the final model. Note that each data point corresponds to a pair of fish, yet we opted not to include the identities of each fish as random effects because it was not possible to structure the random effects such that the intra- and inter-individual variation attributable to fish identities could be accounted for. Instead, group ID was included as a random intercept.

We next adopted a permutation-based approach to directly investigate whether certain dyads of fish engaged in more or fewer contests than expected by chance (note that this analysis does not account for distance between group members). To do this, we obtained a rate of contest behavior for each dyad by calculating the number of contest behaviors that the fish displayed per minute when they had the opportunity to interact (i.e., the time when the fish were not hiding in their shells from predators). We took the difference in average contest behavior rate between related and unrelated dyads to be our observed response and did so for each sex pairing (female–female, female–male, and male–male) separately. To build our null distributions, we permuted the contest behavior rates within each pairing without resampling (i.e., all contest behavior rates were randomized within female–female, female–male, and male–male dyad types), and then took the same difference values between related and unrelated dyads. We conducted 10,000 permutations of the data and calculated *P*-values as the proportions of the null distributions that were at least as extreme as our observed difference values. The permutation tests were performed using a custom R script, which can be found in the Supplementary Materials.

Finally, we investigated whether the spatial arrangement of home shells on a territory reflected the kin relationships among the shell owners. We fit a linear mixed effects model (LMM, using the “glmmTMB” R package, Brooks et al. 2017) and included distance between the home shells for the fish in each dyad as the response variable (cm, log-transformed). We included sex pairing (female–female, female–male, male–male) and kinship assignment (related, unrelated) as predictor variables. The interaction term between the two predictors did not significantly improve model fit, as tested with a likelihood ratio test, and was therefore not included in the final model. Heteroscedasticity in the data was modeled directly using a dispersion formula (Brooks et al. 2017). Group ID was included as a random intercept.

## RESULTS

The 22 *N. multifasciatus* groups in our study contained a total of 70 adult fish, and average group size was 3.1 adult fish (range: 2 to 6), with an average of 1.4 males (1 to 3) and 1.8 females (1 to 4). These represent small to mid-sized groups in this population (average group size in population = 6.3 ± 4.4, Bose, Dabernig-Heinz, et al. 2022; Bose, Koch, et al. 2022). Six of the groups we investigated contained subordinate males in addition to the dominant males (*N* = 9 subordinate males in total). Focusing on groups of these sizes was necessary because of the need to identify each individual in the field videos and match them to their multi-locus genotypes, a task that gets progressively more difficult as group size increases. The fish used in this study could be separated into 24 female–female (cohabiting) dyads, 55 male–female dyads, and 13 male–male dyads. ML-Relate categorized the dyads of cohabiting adults into 67 unrelated and 25 related dyads (see Figure 2A for a breakdown of sample sizes in each category).

**Figure 1:**
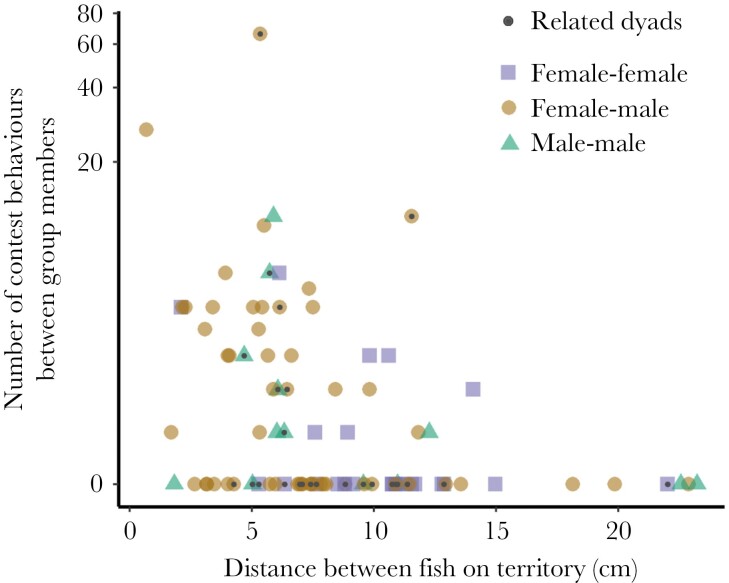
The frequency of contest behaviors between group members, as observed in each field video, decreases with distance separating their home shells. Each colored shape represents a dyad of fish, and the dark dots overlaid represent related dyads of fish. The y-axis is visualized on a log-scale.

**Figure 2:**
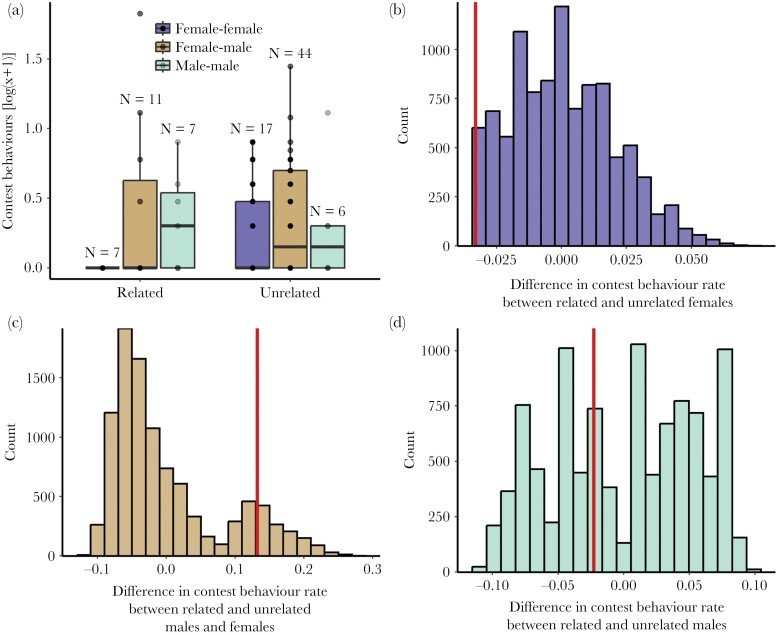
(A) Number of contest behaviors displayed by dyads of cohabiting adult *Neolamprologus multifasciatus* categorized based on the sex of the fish and their genetic relatedness. For visualization purposes, the data shown here are raw counts after a log(x+1) transformation and do not account for the time windows when fish were hiding from predators (see Methods for details). Box plots show medians (horizontal bar), the interquartile range (boxes) and the extent of the data no more than 1.5 times the interquartile range above the box (whiskers). The number of dyads contributing to each group is given above the boxes. (B) Null distribution for the difference in average contest behavior rate between related and unrelated female–female dyads. (C) Corresponding null distribution for female–male dyads. (D) Corresponding null distribution for male–male dyads. The vertical red lines indicate our observed difference values.

The distance separating the home shells of group members was negatively correlated with the frequency of contest behaviors in a dyad (GLMM, estimate ± SE = −3.38 ± 0. 72, z = −4.71, *P* < 0.0001, Figure 1). The sex pairings did not differ significantly from one another with respect to their contest interaction rates (male–male vs. female–female: estimate ± SE = −0.93 ± 0.68, z = −1.37, *P* = 0.17; female–male vs. female–female: estimate ± SE = −0.52 ± 0.45, z = −1.16, *P* = 0.25; male–male vs. female–male, estimate ± SE = −0.41 ± 0.53, z = −0.78, *P* = 0.44). Kinship did not clearly correlate with the expression of contest behaviors when considering sex pairing together (i.e., main effect of kinship, estimate ± SE = 0.11 ± 0.46, z = 0.24, *P* = 0.81).

However, we detected a conspicuous absence of contest behaviors within related dyads of females compared to unrelated dyads, and our permutation approach highlighted this difference in comparison to random chance (*P* = 0.05, Figure 2A,B). While related females did not engage in any contest behavior with each other, unrelated females did (Figure 2A). Note that our observed difference in contest behavior rate between related and unrelated females was the maximum difference observable given all possible permutations of the data. That is, we never observed a more extreme difference in contest behavior rate among related and unrelated females in any of our data permutations (though approximately 5% of the permutations yielded the same difference value, see Figure 2B). Neither male–female contest behavior (*P* = 0.11, Figure 2C), nor male–male contest behavior (*P* = 0.42, Figure 2D) differed clearly between related and unrelated dyads.

**Figure 3:**
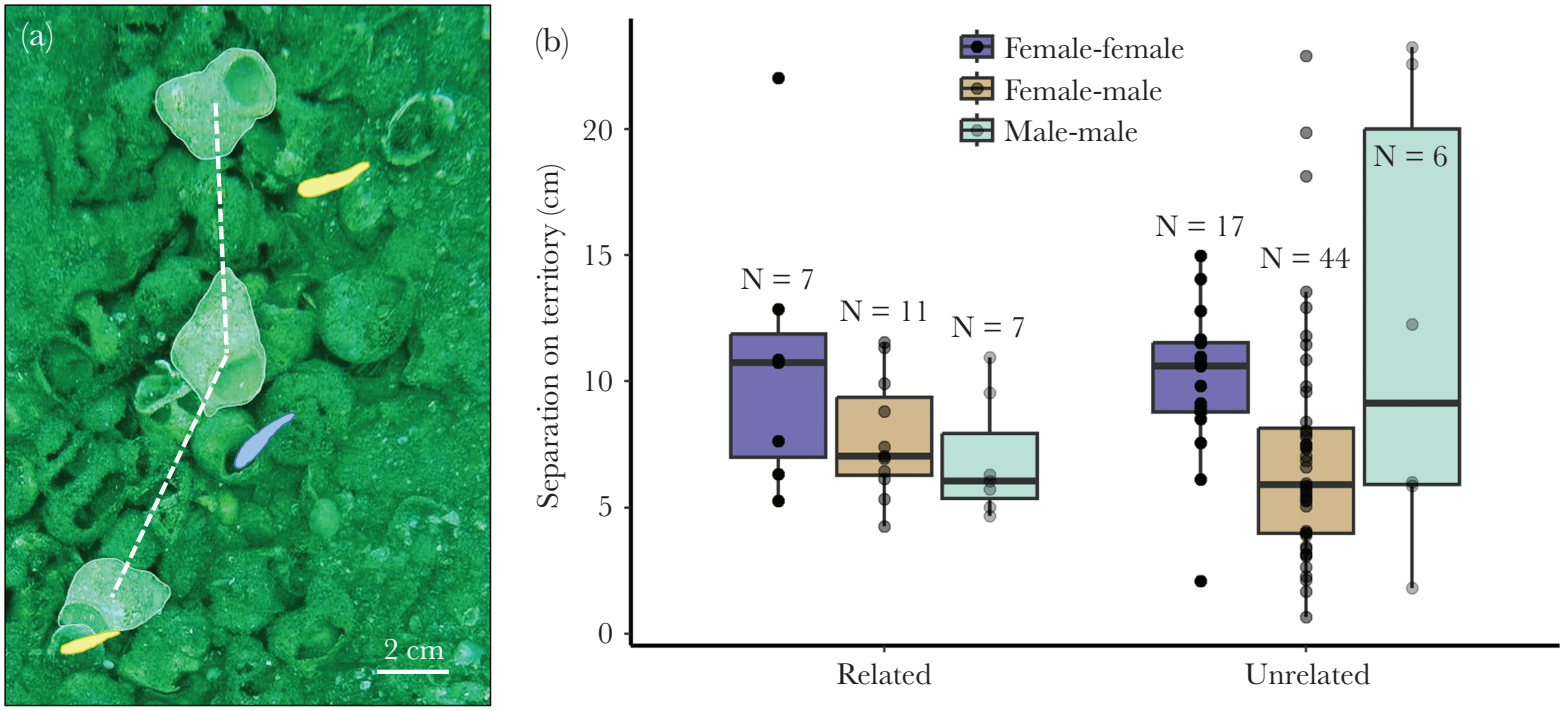
(A) Top-down image of a *Neolamprologus multifasciatus* territory in the wild, with three “home shells” highlighted. The corresponding shell owners are also highlighted, with the dominant male shown in blue and two females in yellow. Dashed white lines indicate how distances between pairs of home shells were measured. (B) Distances separating the home shells of adult *N. multifasciatus* group members. Data are shown by the sex of the fish in each dyad and by their kinship assignment. Box plots show medians (horizontal bar), the interquartile range (boxes) and the extent of the data no more than 1.5 times the interquartile range above the box (whiskers). The number of dyads contributing to each group is given by each box.

The home shells of each group member were spaced on average 8.3 ± 4.7 cm (± SD, range: 0.67–23.2 cm) apart from one another (Figure 3A). On average, females lived further apart from other females on their territory than they did from males (estimate ± SE = 0.17 ± 0.05, z = −3.3, *P* = 0.0001, Figure 3B), and females lived further apart from other females than males lived from other males (estimate ± SE = −0.19 ± 0.07, z = −2.7, *P* = 0.0072). Relatedness did not correlate with the distances at which group members lived from one another (estimate ± SE = −0.06 ± 0.05, z = −1.3, *P* = 0.18). Post hoc analysis of the variation in the data revealed that unrelated dyads of males lived at more variable distances from one another than related dyads of males (dispersion model, estimate ± SE = 2.36 ± 0.81, z = 2.92, *P* = 0.0036, Figure 3B). This analysis was added post hoc because we did not have a priori predictions about variance. Unrelated male–female dyads also lived at more variable distances from one another than related male–female dyads (estimate ± SE = 1.61 ± 0.48, z = 3.33, *P* = 0.0009). The variation in distances at which dyads of females lived from one another, however, did not clearly differ between related and unrelated dyads (estimate ± SE = −0.089 ± 0.64, z = −0.14, *P* = 0.89).

## DISCUSSION

When groups are comprised of both relatives and non-relatives, individuals may sometimes benefit by behaving less competitively with kin (Pizzari et al. 2015). This can be achieved by modulating aggression toward group members according to genetic relatedness, and/or by maintaining physical separation between kin. In this study, we used a social cichlid fish to examine whether kinship mediates the expression of contest behaviors between group members and their spatial positioning on a jointly held territory.

In some social species, aggression toward unrelated group members is higher than toward related group members. For example, in white-nosed coatis, *Nasua narica*, individuals that are unrelated to the majority of their group members receive more aggression than those that are related (Gompper et al. 1997; though Hirsch et al. 2012 did not detect a correlation between aggression and kinship in a related species, the ring-tailed coati, *Nasua nasua*). Tóth et al. (2009) also showed in the house sparrow, *Passer domesticus*, that birds are less likely to aggressively scrounge from the foraging efforts of their close kin than from unrelated individuals. In bonnet macaques, *Macaca radiata*, high-ranking females preferentially show aggression toward lower-ranking non-kin females and their daughters (Silk et al. 1981). In the current study on *N. multifasciatus*, we did not detect any clear correlations between kinship and within-group contests for male–male or female–male dyads. However, we did find an absence of contest behaviors between related females, whereas unrelated females did engage in contests with one another. This was despite similar distances separating related and unrelated female dyads on their territory, thereby ruling out physical separation as an explanation for the lack of contests among related females. There are several possible reasons for why kinship may play a role in governing contest behavior among females but not among males. First, the proportion of same-sex interaction partners that are relatives of an actor is higher for males than for females in *N. multifasciatus* (see “scale of competition,” Frank 1998; Griffin et al. 2004). A recent population genetics study revealed that average male–male relatedness in *N. multifasciatus* groups is ~0.25, far greater than average female–female relatedness, which is near to zero (Bose, Koch, et al. 2022). If local relatives dominate the competitive arena, then this limits the scope for relatedness to select for reduced competitive behavior (West et al. 2001; Griffin et al. 2004), and this might be the case for male*s* more so than females in this species. Second, while males follow a strict hierarchy, with a large dominant male at the top followed by subordinates, females tend to be more equivalent in terms of fighting ability and status. Females occupy individual sub-territories where they can each breed separately, but they also compete with one another over territory space and shells (Bose et al. 2021). Since females use their shells to raise offspring, contests among female kin that result in a loss of territory space for one of them are likely to incur inclusive fitness costs to the other. Overall, this could favor females that focus their aggression toward non-kin rival females in a mixed-kin setting. Breeding females directing their aggression preferentially toward non-kin females is also seen in Columbian ground squirrels, *Urocitellus columbianus* (Viblanc et al. 2016). It is also possible that we did not detect a clear effect of kinship on male–male contest behavior, because male–male interactions *N. multifasciatus* are comprised of two types, namely when two subordinate males vie for a position in the breeding queue, and when dominant males exert aggression toward subordinates. While our study included both dominant and subordinate males, once the kin relationships among them were clarified, we unfortunately did not have the sample sizes needed to test for an effect of kinship among males on subordinate–subordinate and dominant–subordinate contests separately. In the case of subordinate–subordinate interactions within a breeding queue, kin-competition can be fierce, reducing the influence of kinship on aggression, and it can also favor dispersal as a means to avoid the competition entirely (Ridley and Sutherland 2002). It would therefore be valuable for future studies to investigate the social dynamics of dominants and subordinates in groups with greater numbers of males. It is also worth mentioning that the effects of relatedness on male–male aggression have been investigated in other fish species, but without uncovering significant correlations (e.g., three-spined sticklebacks, *Gasterosteus aculeatus* L., Mehlis et al. 2009).

We sampled the spatial structure of the home shells in each *N. multifasciatus* group via a snapshot approach and compared the separation between home shells among different sex and kinship assignments. On average, females lived further apart from one another than they did from males, or than males did from each other (Figure 3B). This is likely due in-part to females defending their sub-territories versus other females thereby maintaining physical separation (Bose et al. 2021; Gübel et al. 2021). In fact, females almost never lived closer than 5 cm to one another, though distances between females and males could often be smaller (Figure 3B). A previous study showed that dominant males can occupy a central position within their territory, which suggests that they are rarely very distant from any other group member (Bose et al. 2021). Since each of our 22 focal groups contained a dominant male, but only several contained subordinates, our results are largely governed by the positioning of dominant males. Across species, dominant individuals are often located in more central, or optimal, positions within groups (Krause 1994; Hall and Fedigan 1997; Hirsch 2007). For example, in Assamese macaques, *Macaca assamensis*, more dominant individuals tend to be located centrally within their groups where predation risk is lower (Heesen et al. 2015), and in meerkats, *Suricata suricatta*, dominant individuals are often located at the forefront of their group where feeding opportunities are higher (Gall and Manser 2018).

Kinship did not clearly correlate with the average distances that group members lived from one another, and this implies that kin and non-kin occupy interspersed positions on their territory, rather than compartmentalized kin regions or kin aggregations (which we would have detected as closer distances among kin than among non-kin). An interspersed distribution of kin and non-kin individuals lessens kin conflict over local resources, which in this case are empty shells situated at the border between sub-territories and whose ownership is less certain. The degree to which multiple individuals can share local resources will likely influence the costs and benefits of clustering together with kin versus interspersing with non-kin. Shells are durable and defendable resources that only one fish can use at a time. This gives shells a high survival and reproductive value, making them attractive objects for individuals to aggressively protect and remove from public use (see “privatization,” Strassmann and Queller 2014). The non-sharable nature of these resources means that kin competition over shells would incur significant inclusive fitness costs, helping to explain why kin apparently intersperse with non-kin in *N. multifasciatus* (i.e., to avoid removing a vital resource from a nearby relative). In contrast, in species that are able to share resources and food within an area, such as southern pied babblers, *T. bicolor*, or Atlantic salmon (juveniles), *Salmo salar* (L.), kin have been shown to aggress less with one another, cluster together in space, and overlap in territory ranges (Griffiths and Armstrong 2002; Humphries et al. 2021).

Unrelated dyads of fish, particularly those involving males (i.e., male–male, male–female dyads), lived at more variable distances than related dyads. One explanation for these patterns that warrants future testing is if subordinate males that are unrelated to the dominant male are relegated to the outermost periphery of their group’s territory. Such positioning could imply that these outer individuals have less contact with more central group members, as the opportunity for social behavior declines with distance between home shells (Figure 1; Gübel et al. 2021). If non-kin subordinate males located on the periphery have fewer opportunities to interact with other group members, but when they do interact it is frequently agonistic, then this may also mask an effect of kinship in modulating contests among males. The *N. multifasciatus* groups used in this study represent small to medium-sized groups in the wild. In order to evaluate whether subordinate males that are unrelated to the dominant are indeed more peripheral than subordinate males that are related to the dominant, a future study will be needed that examines larger social groups, which contain more subordinate males and occupy more space.

In summary, we found that the *N. multifasciatus* females in our study refrained from engaging in contest behaviors with cohabiting females that were also their kin, whereas they did engage in contests with non-kin females. This was despite the fact that the kin and non-kin females lived at similar distances from one another on their group’s territory. We did not find clear evidence of less aggression toward kin within male–male or male–female dyads, and these dyads lived at more variable distances from one another on the territory. We suggest that these latter patterns may be explained by non-kin subordinate males residing in more peripheral locations on the territory, where they would have fewer opportunities to interact with other group members. However, future work examining larger *N. multifasciatus* groups with greater numbers of subordinate males will be necessary to verify this idea. Overall, our study suggests that aggression among group members can be mediated by their relatedness, that these patterns can differ between the sexes, and that spatial relationships can play an important role in determining the extent to which group members compete with one another.

## Supplementary Material

arad036_suppl_Supplementary_Material_S1

arad036_suppl_Supplementary_Material_S2

arad036_suppl_Supplementary_Material_S3

## Data Availability

Analyses reported in this article can be reproduced using the data provided by Bose et al. (2023).

## Notes

We are grateful to the Department of Fisheries in Mpulungu, Zambia for supporting our research at Lake Tanganyika. We also thank the staff at the Tanganyika Science Lodge for their hospitality and our guides and boat drivers for their incredibly hard work.
