# Supplementary material for: Aggression and spatial positioning of kin and non-kin fish in social groups
Source: Behav Ecol. 2023 May 12;34(4):673–81. doi: 10.1093/beheco/arad036 (PMC10332448; doi:10.1093/beheco/arad036)
Supplement: arad036_suppl_Supplementary_Material_S1 [file arad036_suppl_supplementary_material_s1.docx]

SUPPLEMENTARY MATERIALS

**Aggression and spatial positioning of kin and non-kin fish in social groups**

Aneesh P. H. Bose, Johanna Dabernig-Heinz, Jan Oberkofler, Lukas Koch, Jacqueline Grimm, Kristina M. Sefc, Alex Jordan

**Supplementary Materials Table S1:** Marker polymorphism of 20 microsatellites used in this study based on reference population. k: Number of alleles. N: Number of individuals genotyped at the particular locus. H_Obs_: Observed Heterozygosity (proportion of heterozygotes at this locus). H_Exp_: Expected heterozygosity (expected proportion of heterozygotes given allele frequencies). HW: Adherence to Hardy-Weinberg Equilibrium, tested in CERVUS using a Bonferroni correction (Bonferroni corrected α = 0.0025). Shading denotes separation of markers into different multiplexes.

| **Locus** | **k** | **N** | **H_Obs_** | **H_Exp_** | **HW *P*-value** | **Conc. in primer mix (pmol/μL)** | **Reference** |
| --- | --- | --- | --- | --- | --- | --- | --- |
| Multiplex 1 |  |  |  |  |  |  |  |
| Pmv17 | 19 | 233 | 0.906 | 0.912 | 0.50 | 0.5 | (Crispo et al. 2007) |
| UNH890 | 6 | 232 | 0.414 | 0.436 | 0.73 | 1.0 | (Carleton et al. 2002) |
| UNH908 | 25 | 235 | 0.843 | 0.875 | 0.36 | 3.0 | (Carleton et al. 2002) |
| Gm634 | 15 | 234 | 0.799 | 0.818 | 0.42 | 1.0 | (Lee et al. 2005) |
| Ppun9 | 21 | 233 | 0.674 | 0.748 | 0.02 | 0.5 | (Taylor et al. 2002) |
| Hchi59 | 17 | 232 | 0.845 | 0.864 | 0.52 | 1.0 | (Maeda et al. 2008) |
| UNH216 | 11 | 232 | 0.603 | 0.584 | 0.83 | 4.0 | (Lee and Kocher 1996) |
| UME002 | 7 | 228 | 0.61 | 0.627 | 0.44 | 4.0 | (Parker and Kornfield 1996) |
| Multiplex 2 |  |  |  |  |  |  |  |
| Pmv3 | 31 | 237 | 0.768 | 0.775 | 0.04 | 1.0 | (Crispo et al. 2007) |
| GM264 | 17 | 234 | 0.85 | 0.859 | 0.42 | 4.0 | (Lee et al. 2005) |
| Ppun5 | 23 | 233 | 0.695 | 0.722 | 0.19 | 3.0 | (Taylor et al. 2002) |
| TmoM13 | 25 | 234 | 0.829 | 0.907 | 0.44 | 4.0 | (Zardoya et al. 1996) |
| TmoM25 | 4 | 231 | 0.732 | 0.671 | 0.55 | 2.0 | (Zardoya et al. 1996) |
| Hchi36 | 4 | 230 | 0.539 | 0.559 | 0.44 | 1.0 | (Maeda et al. 2008) |
| UME003 | 17 | 232 | 0.897 | 0.869 | 0.56 | 2.0 | (Parker and Kornfield 1996) |
| Multiplex 3 |  |  |  |  |  |  |  |
| TmoM11 | 7 | 234 | 0.667 | 0.677 | 0.87 | 1.5 | (Zardoya et al. 1996) |
| UNH2075 | 19 | 233 | 0.773 | 0.77 | 0.23 | 2.5 | (Albertson et al. 2003) |
| NP101 | 18 | 232 | 0.81 | 0.749 | 0.02 | 3.5 | (Brandtmann et al. 1999) |
| Pzeb4 | 8 | 232 | 0.612 | 0.61 | 0.98 | 2.0 | (Van Oppen et al. 1997) |
| UNH974 | 33 | 219 | 0.863 | 0.926 | 0.60 | 4.0 | (Carleton et al. 2002) |

**
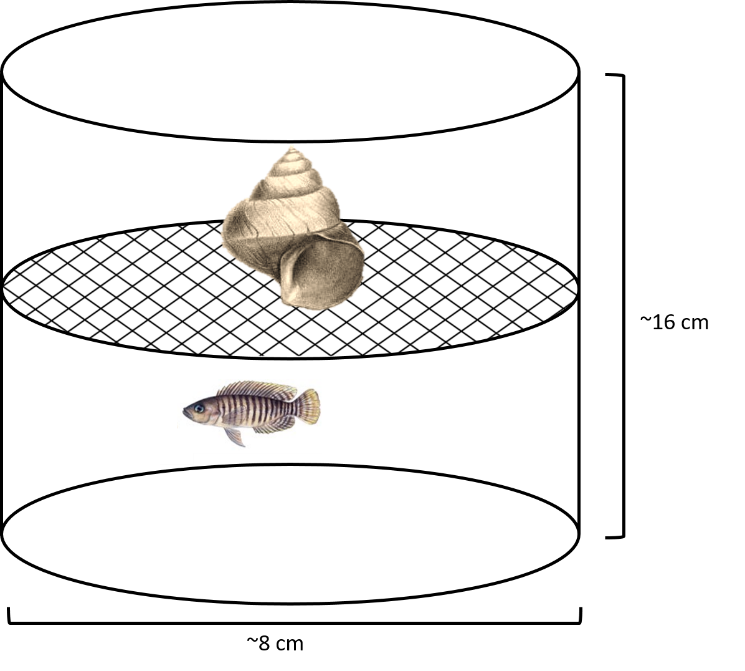
**

**Supplementary Materials Figure S1:** Diagram of passive fish extraction chamber, used to remove *Neolamprologus multifasciatus* from their shells while underwater without harm or handling stress to the animal. The top and bottom halves of the transparent chamber are detachable and are separated by a mesh. Shells containing fish are placed in the top half of the chamber on top of the mesh. Within a period of time (usually 30-45 minutes, but sometimes longer, AB personal observations), the fish will emerge from the shell and swim to the bottom chamber. The fish can then be removed from the bottom chamber and used in a field study or experiment.

**
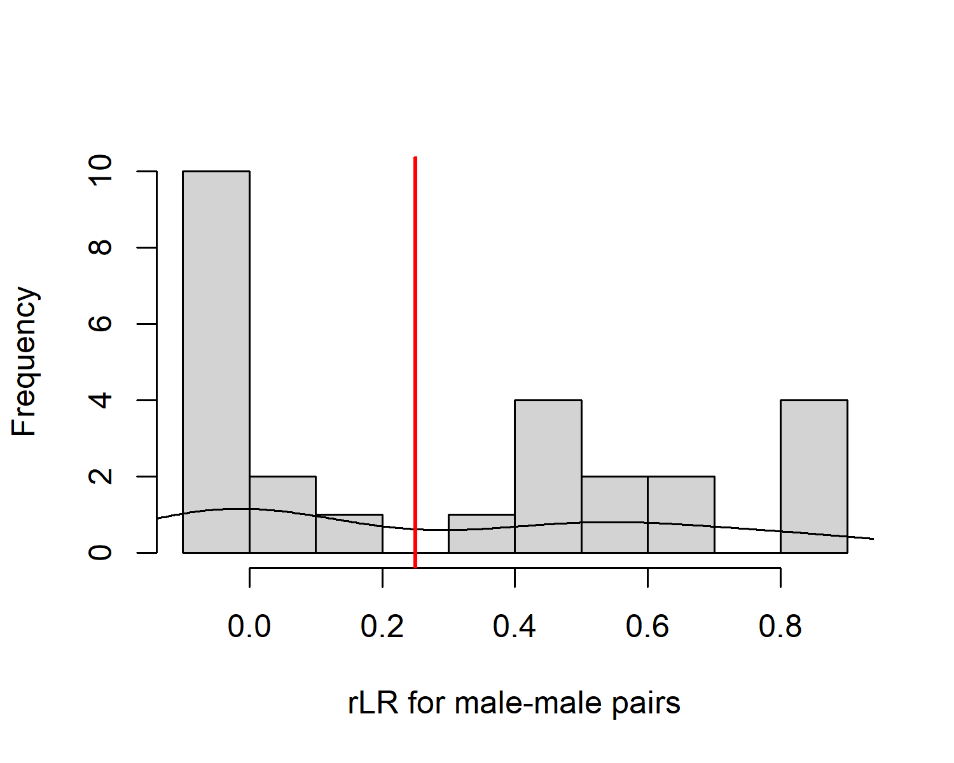
**

**Supplementary Materials Figure S2:** Relatedness among male group members (calculated using the Lynch-Ritland estimator, Lynch and Ritland 1999) follows a bimodal distribution. Data are shown as a histogram and as a density plot. R = 0.25, denoting the expected average relatedness between half-siblings, is shown with a red line.

**
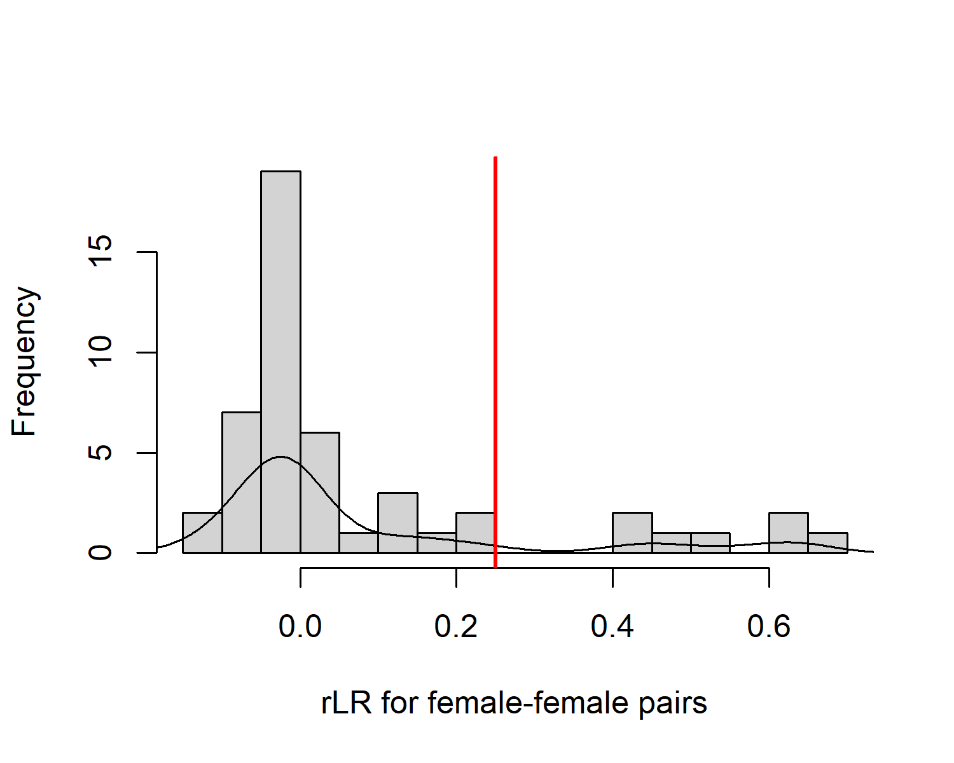
**

**Supplementary Materials Figure S3:** Relatedness among female group members (calculated using the Lynch-Ritland estimator, Lynch and Ritland 1999) follows a bimodal distribution. Data are shown as a histogram and as a density plot. R = 0.25, denoting the expected average relatedness between half-siblings, is shown with a red line.

**
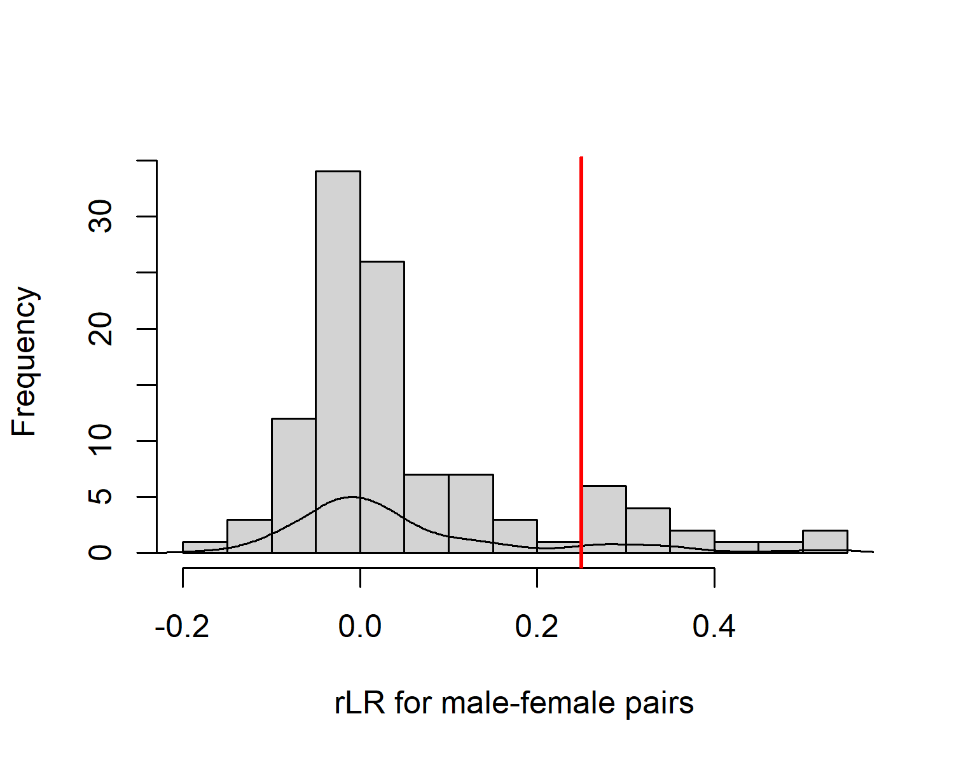
**

**Supplementary Materials Figure S4:** Relatedness between opposite sex group members (calculated using the Lynch-Ritland estimator, Lynch and Ritland 1999) follows a bimodal distribution. Data are shown as a histogram and as a density plot. R = 0.25, denoting the expected average relatedness between half-siblings, is shown with a red line.

REFERENCES

Albertson RC, Streelman JT, Kocher TD. 2003. Directional selection has shaped the oral jaws of Lake Malawi cichlid fishes. Proc Natl Acad Sci U S A. 100(9):5252–5257.

Brandtmann G, Scandura M, Trillmich F. 1999. Female-Female Conflict in the Harem of a Snail Cichlid (*Lamprologus ocellatus*): Behavioural Interactions and Fitness Consequences. Behaviour. 136(9):1123–1144.

Carleton KL, Streelman JT, Lee BY, Garnhart N, Kidd M, Kocher TD. 2002. Rapid isolation of CA microsatellites from the tilapia genome. Anim Genet. 33(2):140–144.

Crispo E, Hagen C, Glenn T, Geneau G, Chapman LJ. 2007. Isolation and characterization of tetranucleotide microsatellite markers in a mouth-brooding haplochromine cichlid fish (*Pseudocrenilabrus multicolor victoriae*) from Uganda. Mol Ecol Notes. 7(6):1293–1295.

Lee BY, Lee WJ, Streelman JT, Carleton KL, Howe AE, Hulata G, Slettan A, Stern JE, Terai Y, Kocher TD. 2005. A second-generation genetic linkage map of tilapia (*Oreochromis* spp.). Genetics. 170(1):237–244.

Lee WJ, Kocher TD. 1996. Microsatellite DNA markers for genetic mapping in *Oreochromis niloticus*. J Fish Biol. 49(1):169–171.

Lynch M, Ritland K. Estimation of pairwise relatedness with molecular markers. Genetics. 1999;152:1753–66.

Maeda K, Takeshima H, Mizoiri S, Okada N, Nishida M, Tachida H. 2008. Isolation and characterization of microsatellite loci in the cichlid fish in Lake Victoria, *Haplochromis chilotes*. Mol Ecol Resour. 8(2):428–430.

Van Oppen MJH, Rico C, Deutsch JC, Turner GF, Hewitt GM. 1997. Isolation and characterization of microsatellite loci in the cichlid fish *Pseudotropheus zebra*. Mol Ecol. 6(4):387–388.

Parker A, Kornfield I. 1996. Polygynandry in Pseudotropheus zebra, a cichlid fish from Lake Malawi. Environ Biol Fishes. 47(4):345–352.

Taylor MI, Meardon F, Turner G, Seehausen O, Mrosso HDJ, Rico C. 2002. Characterization of tetranucleotide microsatellite loci in a Lake Victorian, haplochromine cichlid fish: A *Pundamilia pundamilia* x *Pundamilia nyererei* hybrid. Mol Ecol Notes. 2(4):443–445.

Zardoya R, Vollmer DM, Craddock C, Streelman JT, Karl S, Meyer A. 1996. Evolutionary conservation of microsatellite flanking regions and their use in resolving the phylogeny of cichlid fishes (Pisces: Perciformes). Proc R Soc B Biol Sci. 263(1376):1589–1598.
